# Eosinophil-cationic protein - a novel liquid prognostic biomarker in melanoma

**DOI:** 10.1186/s12885-019-5384-z

**Published:** 2019-03-07

**Authors:** Annika Krückel, Alvaro Moreira, Waltraud Fröhlich, Gerold Schuler, Lucie Heinzerling

**Affiliations:** Department of Dermatology, University Hospital Erlangen, Friedrich-Alexander-University Erlangen-Nürnberg (FAU), 91054 Erlangen, Germany

**Keywords:** Eosinophil cationic protein (ECP), Melanoma, Biomarker, Prognosis, Eosinophils

## Abstract

**Background:**

The role of eosinophils in cancer is not yet completely understood, but patients with eosinophilia show a trend towards longer survival in several types of cancer, including melanoma. However, eosinophil count at initial diagnosis of metastatic melanoma does not predict survival. Since eosinophil cationic protein (ECP) mediates anticancer effects, such as tissue remodelling and cytotoxic activity, we investigated this marker as an early prognostic marker in metastatic melanoma.

**Methods:**

Serum of 56 melanoma patients was collected at the time of diagnosis of metastatic disease. ECP levels as measured by ELISA were correlated with overall survival (OS) in patients before systemic therapy with immunotherapy or chemotherapy. Statistical analyses were performed using the Log–Rank (Mantel–Cox) test.

**Results:**

The median OS for patients with high serum ECP above 12.2 ng/ml was 12 months (*n* = 39), compared to 28 months for patients with ECP below this threshold (*n* = 17; *p* = 0.0642). In patients with cutaneous melanoma, excluding patients with uveal and mucosal melanoma, the survival difference was even more striking (*p* = 0.0393). ECP’s effect size on OS was observed independently of the consecutive therapy. ECP levels were not correlated with LDH levels.

**Conclusion:**

ECP seems to be a novel prognostic serum marker for the outcome of melanoma patients, which is independent of LDH and easy to perform in clinical practice. The striking negative prognostic value of high ECP level is unanticipated and can guide patient management.

**Electronic supplementary material:**

The online version of this article (10.1186/s12885-019-5384-z) contains supplementary material, which is available to authorized users.

## Background

Established prognostic markers in melanoma – besides TNM stage – include LDH (lactate dehydrogenase) and performance status [1, 2] while the tumour markers S100 B protein and protein melanoma-inhibitory-activity (MIA) are mostly used to detect progression of disease but do not correlate directly with prognosis [3, 4].

Several studies have shown that eosinophil levels are linked with prognosis in different tumour entities [5–7]. Increased frequencies of eosinophils were described to predict a better outcome in primary small cell oesophageal carcinoma and gastrointestinal, colorectal, breast and prostate cancer [5, 8, 9]. However, patients with eosinophilia show a worse prognosis in other tumour entities such as Hodgkin’s lymphoma, oral squamous cell carcinoma or cervical carcinoma [5, 6, 9–12]. Due to these inconsistent findings the role of eosinophils in tumour control is still not fully understood [7, 9, 13].

Eosinophil count has already been shown to be a predictive biomarker for therapy with immune checkpoint inhibitors in melanoma [14]. Baseline frequencies as well as an increase of the number of eosinophils between the first and the second infusion of the anti-CTLA-4 antibody ipilimumab correlate with a better overall survival (OS) [14–16]. Regarding therapy with anti-PD-1 antibodies, eosinophil count at baseline also correlated with OS of melanoma patients [14, 17]. Additionally, recent studies by our research group revealed the prognostic value of eosinophils in melanoma patients [9]. A prolonged survival was demonstrated in both cohorts of melanoma patients with eosinophilia, immunotherapy-naive and in patients receiving immunotherapy [9]. However, in most cases patients only developed eosinophilia during the course of metastatic disease, thus eosinophil count at initial diagnosis of metastatic disease did not predict survival [9, 18].

Murine studies indicate that eosinophils are involved in CD8^+^ T cell-mediated tumour rejection by producing chemoattractants, such as CCL5, CXCL9 and CXCL10 [19]. Furthermore, studies on cancer patients also suggest that eosinophilic granulocytes affect tumour cells directly through the secretion of cytotoxic proteins [7, 19].

Eosinophil-derived neurotoxin (EDN), for example, is associated with intratumoural cell apoptosis [7], but the role of other eosinophilic cytotoxins, like eosinophil cationic protein (ECP), eosinophil peroxidase (EPO) or major basic protein (MBP) is not clear yet [7, 20].

ECP serves as a ribonuclease and belongs to RNase A family 3 [7, 12]. Its release can be induced by immunoglobulins (IgE, IgG), surface-bound complement as well as lipid mediators (lipopolysaccharides (LPS) or Lipid A) [7, 12, 21]. Though ECP’s ribonucleolytic activity is low, its cell membrane binding mediates a multitude of further functions, like osmotic lysis, synthesis of reactive oxygen species, reversed membrane asymmetry, chromatin condensation as well as increased Caspase-3-like activity and, thus, cytotoxicity as shown in mammalian cell culture models [22]. It was suggested that ECP might, aside from harming various microorganisms [7, 12, 22, 23], also have cytotoxic activity against cancer cells, such as Hodgkin lymphoma and colorectal tumor cells [7, 12, 24–26], however, its definite role in human cancer is yet to be investigated. In studies on oral squamous carcinoma cell lines, ECP did not just limit cell survival, but induced morphological transformation, including vacuolation, formation of blebs and disabled cell adhesion [24]. On the contrary, ECP was suggested to promote tumour infiltration through muscle fiber corrosion [7]. According to in vitro experiments, ECP was involved in degradation of membrane-associated cytoskeletal proteins and myofibrillar proteins, such as myosin heavy chain as well as α-actin [7]. Moreover, in vitro studies showed that ECP inhibits immune functions such as the production of immunoglobulins as well as T cell proliferation [7, 27]. However, the role of ECP in vivo has to be determined.

This study investigates whether serum levels of ECP have a prognostic value for patients with metastatic melanoma.

## Methods

### Patients and clinical characteristics

In total, 56 patients with metastatic melanoma treated in our clinic from January 2004 to September 2017 were included in this study and analysed retrospectively. Variables that were analysed include gender, age, tumour involvement, type of melanoma, systemic therapies, and overall survival. The patient cohort incorporated patients independently of their subsequent therapy. The patient characteristics are depicted in Table 1, with *p*-values included. The cohort included all histological types of melanoma (cutaneous melanoma, mucosal melanoma, uveal melanoma and melanoma of unknown primary). Due to biological differences between mucosal/uveal and cutaneous melanoma, overall survival (OS) of patients with cutaneous melanoma was analysed separately. Melanoma of unknown primary (MUP) was subsumed under cutaneous melanoma. Blood sera routinely assessed for tumour markers were used for analysis of eosinophil cationic protein (ECP). This retrospective study was exempt from full application to the Ethics Committee, University of Erlangen.

**Table 1:** Patient characteristics

Variables		Patients with ECP high^a^ (*n* = 34)		Patients with ECP low^b^ (*n* = 22)		*p*-value
Age	Median (Range)	60 years (40–83 years)		64 years (31–90 years)		0.3877
OS	Median (Range)	12 months (2–48 months)		28 months (3–44 months)		0.0916
		n	%	n	%	
Gender	Male	22	65	14	64	0.9350
	Female	12	35	8	36	
Therapy	Surgery	31	91	19	86	0.6701
	Interferon-alpha	13	38	7	32	0.6245
	Chemotherapy	29	85	13	59	0.0270
	Radiotherapy	25	74	17	77	0.7520
	Signal transduction inhibitor (targeted therapy)	21	62	9	41	0.1264
	Checkpoint inhibitor therapy (immunotherapy)	9	26	18	82	< 0.0001
Type of melanoma	Cutaneous	20^c^	59	12	55	0.7520
	MUP	5	15	9	41	0.0270
	Uveal	3	9	1	5	> 0.9999
	Mucosal	7^c^	21	0	0	0.0349
LDH	> ULN	20	59	18	82	0.0868
	< ULN	14	41	4	18	
BRAF status^d^	V600 mutation	13	38	9^e^	41	0.7151
	V600 wildtype	14	41	12^e^	55	
Brain metastases (M1d)		18	53	8	36	0.2244

^a^defined as > 16.0 ng/ml

^b^defined as < 16.0 ng/ml

^c^one patient has both, cutaneous and mucosal melanoma

^d^BRAF status of 7 patients was not tested, BRAF test result of one patient was not evaluable

^e^one patient has discrepant test results of two distinct metastases one with V600E mutation and one with wildtype

### Determination of ECP in serum

For determination of ECP blood sera stored at − 20 °C were thawed and measured by enzyme-linked immunosorbent assay ELISA (Cusabio #CSB-E11729h) with a detection range of 1.56–100 ng/ml according to manufacturer’s protocol. Sera from the time at initial diagnosis of metastatic melanoma were taken defining the initial diagnosis as 0–6 months from the date of stage IV diagnosis. Duplicates of each sample were measured. Serum levels of at least 16.0 ng/ml were defined as elevated, because healthy individuals have a 95% range from 2.3–15.9 ng/ml in the serum [28]. Additionally, a cut-off of 12.2 ng/ml, as determined by recursive partitioning, was analysed and correlated with survival.

### Determination of LDH and blood counts

Serum lactate dehydrogenase (LDH) and blood counts were routinely measured in our lab. LDH was analysed by means of the LDH-ratio (actual value divided by the upper limit of normal). Eosinophilia was defined as at least 5% eosinophils in peripheral blood counts.

In 4 of 56 cases eosinophil counts were not analysed at the time of serum assessment. In these patients the eosinophil counts closest to the serum assessment was taken i.e. a maximum of 4 months before or after.

### Statistical methods

Event-time distributions were estimated with the Kaplan-Meier method. Log–Rank (Mantel–Cox) test was performed to determine the *p*-value. Cut-off values were determined with recursive partitioning. For contingency analyses Chi-square test and Fisher’s exact test were utilised. Mann-Whitney test was used for nonparametric tests. Univariate and multivariate analysis were performed with Cox proportional hazard models. Graphing was created using GraphPad Prism and R.

## Results

### ECP is inversely correlated with survival

**Fig. 1:**
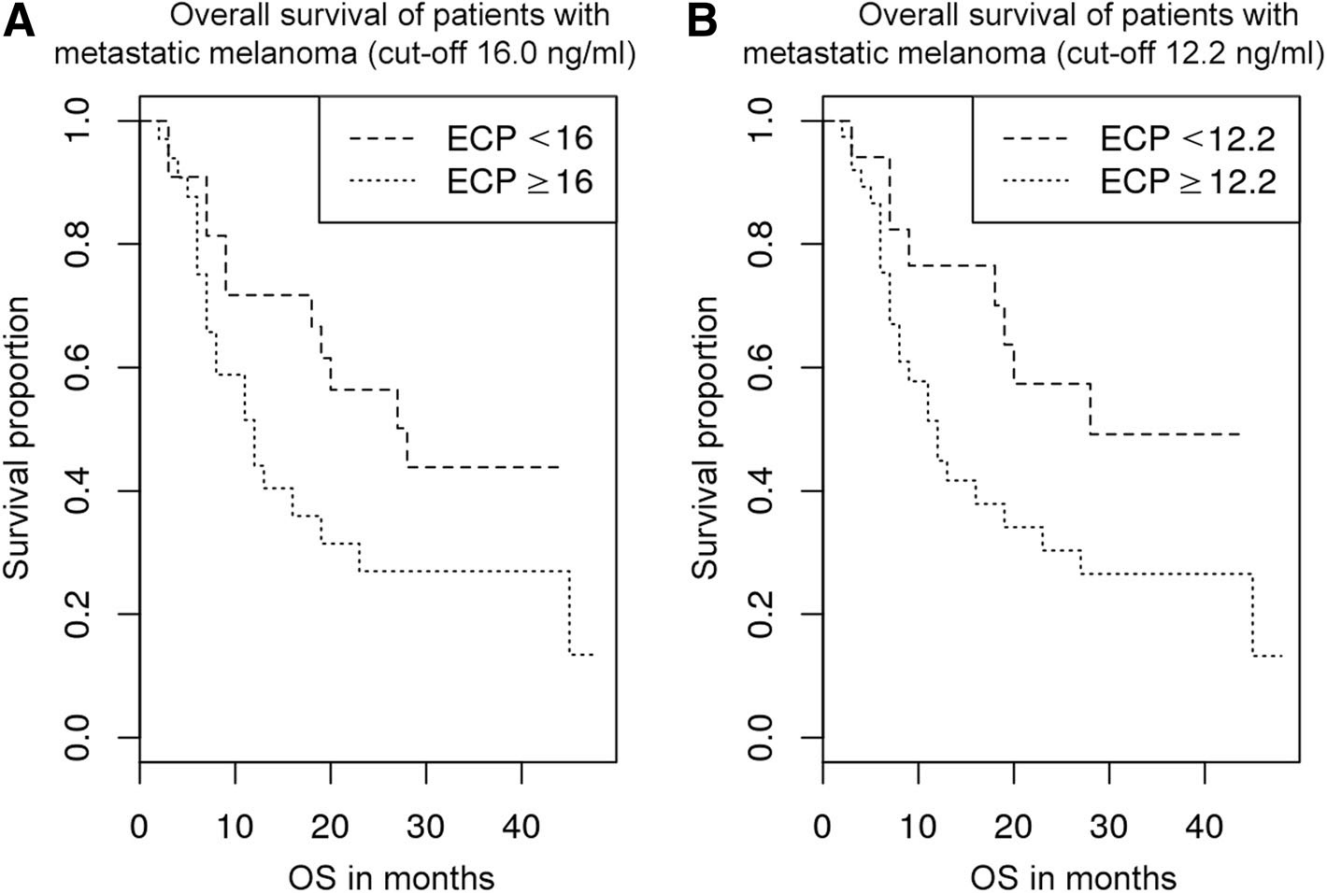
**a** Overall survival of patients with metastatic melanoma (cut-off 16.0 ng/ml). Overall survival of melanoma patients depending on ECP level (*n* = 56). Patients with an ECP level of 16.0 ng/ml or greater (*n* = 34) lived shorter (median OS = 12 months) than patients with less than 16.0 ng/ml serum ECP (*n* = 22; median OS = 28 months; *p* = 0.0916) at the time of metastatic disease (stage 4). **b** Overall survival of patients with metastatic melanoma (cut-off 12.2 ng/ml). Overall survival of patients with an ECP level of 12.2 ng/ml or greater (*n* = 39; median OS = 12 months) and of patients with less than 12.2 ng/ml serum ECP (*n* = 17; median OS = 28 months) at the time of stage 4 diagnosis within all melanoma patients (*n* = 56; *p* = 0.0642)

**Fig. 2:**
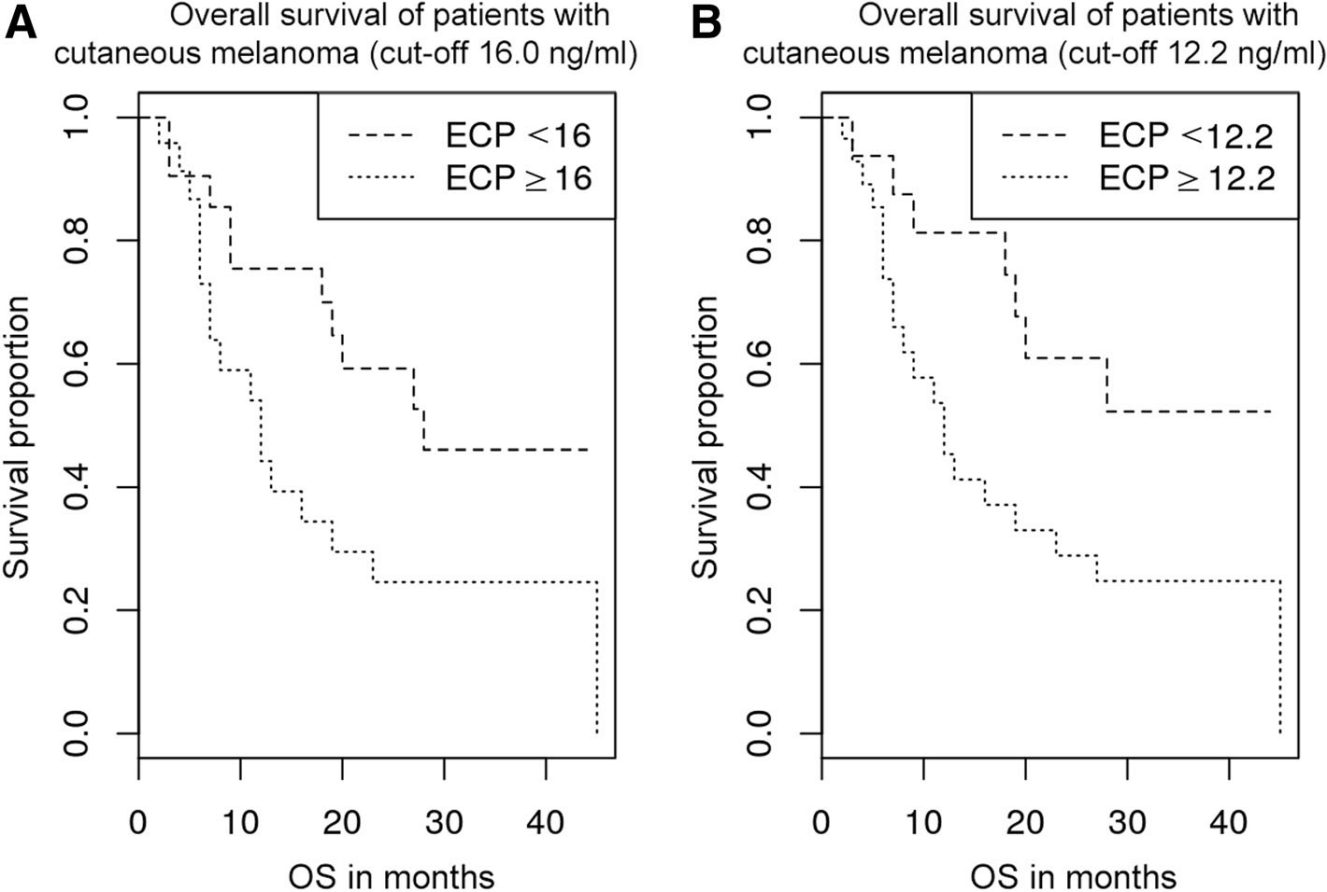
**a** Overall survival of patients with cutaneous melanoma (cut-off 16.0 ng/ml). Overall survival of patients with cutaneous melanoma depending on ECP level (*n* = 45). Patients with an ECP level of 16.0 ng/ml or greater (*n* = 24) lived shorter (median OS = 12 months) than patients with less than 16.0 ng/ml serum ECP (*n* = 21; median OS = 28 months; *p* = 0.0597) at the time of metastatic disease (stage 4). **b** Overall survival of patients with cutaneous melanoma (cut-off 12.2 ng/ml). Overall survival of patients with an ECP level of 12.2 ng/ml or greater (*n* = 29; median OS = 12 months) and of patients with less than 12.2 ng/ml serum ECP (*n* = 16; median OS not reached) at the time of stage 4 diagnosis within patients having cutaneous melanoma (*n* = 45). The difference is statistically significant at *p* = 0.0393

Patients with low ECP at initial diagnosis of metastatic disease had a longer survival in comparison with patients with high ECP. With a cut-off at 16.0 ng/ml serum ECP, the median OS for patients with ECP levels of at least 16.0 ng/ml (*n* = 34) was 12 months, compared with 28 months for patients with levels below this threshold (*n* = 22; *p* = 0.0916; Fig. 1a). A cut-off value of 16.0 ng/ml was used since 95% of healthy individuals have ECP values in the range from 2.3–15.9 ng/ml in the serum [28]. Dichotomizing at 12.2 ng/ml, patients with higher serum levels (*n* = 39) had a median OS of 12 months, compared with 28 months for patients below this threshold (*n* = 17; *p* = 0.0642; Fig. 1b). In a subsequent analysis the subgroup of uveal/mucosal melanoma with their different biology was excluded. The remaining patients with cutaneous melanoma (*n* = 45) were analysed separately. Using a cut-off of 16.0 ng/ml, patients with elevated ECP levels (*n* = 24) had a median OS of 12 months, compared with 28 months for patients below this threshold (*n* = 21; *p* = 0.0597; Fig. 2a). Dichotomizing at 12.2 ng/ml, patients with lower ECP levels (*n* = 16) showed a statistically significant longer OS (median OS not reached) than patients with higher ECP levels (median OS 12 months; *n* = 29; *p* = 0.0393; Fig. 2b). Uveal and mucosal melanoma patients were not analysed separately due to the small number of subjects, with 4 and 7 cases, respectively. Interestingly, 3 out of 4 uveal melanoma patients and all mucosal melanoma patients (7/7) were ECP high (cut-off: 16.0 ng/ml).

### ECP and eosinophilia

In order to investigate whether occurrence of eosinophilia (defined as at least 5% eosinophils in peripheral blood) was linked to presence of ECP, eosinophil counts in the course of the metastatic disease were analysed and compared to ECP serum levels. From the patients with decreased ECP serum levels (defined as < 16.0 ng/ml, *n* = 22) at diagnosis of metastatic disease, only 9% (*n* = 2) had eosinophilia at that time. Regarding the patients with increased ECP levels (*n* = 34), eosinophilia was also present in only 15% (*n* = 5) of the patients.Within the patients with initially increased ECP levels, 47% (16/34) had at some point during the course of their disease an eosinophilia, whereas 32% (7/22) of the patients with decreased ECP also experienced one. In 4 of these 7 patients, blood sera at time of occurrence of eosinophilia could be analysed as well. Interestingly, 75% (3/4) of them still had decreased ECP levels when eosinophilia peak was reached (average ECP value = 6.1 ng/ml). Only in one patient, ECP serum levels exceeded the cut-off (defined as 16.0 ng/ml), but still remained lower (35.7 ng/ml) than the average value of ECP levels of the patients with increased levels at diagnosis of metastatic disease (46.8 ng/ml, *n* = 34).

Regarding development of eosinophilia after ECP assessment, no significant difference (*p* = 0.2824) could be found between patients with decreased ECP values and patients with increased ECP values.

### ECP is independent of LDH

In order to investigate the role of ECP as an independent biomarker, we compared its serum levels in all patients of our cohort with serum lactate dehydrogenase (LDH) values, also taken at initial diagnosis of metastatic disease. As our statistical analysis shows, serum levels of ECP are independent of serum lactate dehydrogenase (Additional file 1). Coefficient of determination r2 was 0.026, meaning only 2.6% of the variation on ECP levels can be explained by variations of LDH levels.

### Treatments of the cohort

Subgroups of ECP low and ECP high were balanced with respect to age and gender. Treatments of the two cohorts were comparable (radiotherapy, surgery, interferon-alpha) whereas differences were found in the frequency of chemotherapy, immunotherapy and signal transduction therapy (Table 1).

### ECP as an independent prognostic biomarker

In order to identify potentially confounding variables, Cox proportional hazard models were used for univariate and multivariate analysis. Age and gender had no influence on effect (Table 2). High ECP (cut-off: 16 ng/ml) increases the risk for a death, whereas targeted therapy as well as immunotherapy decrease this risk. The effect size of the influence of high ECP on survival is diminished by subsequent treatment with targeted or immunotherapy, but remains above 1 across all models and thus represents a marker for a decrease of OS.

**Table 2:** Adjusting for gender, age, targeted and immunotherapy (multivariate analysis)

	(1)	(2)	(3)	(4)
ECP > 16	1.869* (0.893, 3.913)	1.869* (0.893, 3.913)	1.873* (0.895, 3.918)	1.300 (0.578, 2.922)
gender		0.999 (0.482, 2.074)	0.987 (0.475, 2.053)	0.692 (0.311, 1.538)
age			1.006 (0.977, 1.035)	1.008 (0.978, 1.039)
Targeted therapy				0.728 (0.318, 1.663)
Immunotherapy				0.327** (0.130, 0.826)
Observations	56	56	56	56
R^2^	0.050	0.050	0.053	0.146
Max. Possible R^2^	0.982	0.982	0.982	0.982
Log Likelihood	− 110.967	−110.967	−110.893	−107.992
Wald Test	2.760* (df = 1)	2.760 (df = 2)	2.900 (df = 3)	7.910 (df = 5)
LR Test	2.894* (df = 1)	2.894 (df = 2)	3.044 (df = 3)	8.846 (df = 5)
Score (Logrank) Test	2.841* (df = 1)	2.841 (df = 2)	2.985 (df = 3)	8.503 (df = 5)

Note: *p < 0.1; **p < 0.05

## Discussion

This study suggests that ECP represents an independent novel prognostic biomarker in patients with metastatic melanoma. Patients with higher ECP levels at the time of diagnosis of metastatic disease have a shorter survival compared to patients with lower ECP serum levels. Remarkably, survival difference was even more distinct in the subgroup of patients with cutaneous melanoma or MUP, excluding uveal and mucosal melanoma with their different biology. Furthermore, nearly all patients with uveal and mucosal melanoma showed elevated ECP levels.

Interestingly, ECP was associated with a worse outcome although it is secreted by eosinophils [7] whose presence is positively correlated with OS in melanoma [9]. This reverse correlation with survival was observed irrespective of the kind of therapy the patients were receiving and irrespective of the presence of eosinophilia. ECP’s reported cytotoxicity against cancer cells in vitro [7, 12, 24–26] thus does not correspond to its disadvantageous role in melanoma patients in vivo. Only Pereira et al. hypothesized that ECP might promote tumour progression by supporting tumour infiltration of human muscle tissue [7]. Given this unexpected finding of correlation of ECP high with poorer survival further studies in in vitro models and patient cohorts are required to characterize the role of ECP.

Since ECP is a granule cytotoxic protein of eosinophils [7], we would have assumed that eosinophilia precedes high ECP serum levels. However, this was not the case in our cohort. Only 15% of patients with elevated ECP show eosinophilia compared to 9% with low ECP. In the course of metastatic disease, 32% of patients with low ECP and 47% of patients with elevated ECP experienced eosinophilia. Thus, elevated levels of ECP are not directly associated with the presence of eosinophilia.

Furthermore, there was no correlation of ECP with LDH. Both, age and gender did not influence ECP’s effect size on OS. Importantly, all ECP values were measured at diagnosis of stage IV metastatic disease. It is unclear why the ECP low group was subsequently treated with immunotherapy more often. They were however treated less often with targeted therapy. Targeted as well as immunotherapy, as life prolonging therapies, diminish ECP’s effect size on OS, but high ECP levels still increase the risk of death when adjusting for those variables. Possibly, there is a latent variable that is connected to both, ECP and therapy, which has to be further investigated.

Although the function of ECP in cancer progression or rejection is still not fully understood, its levels were associated with prognosis in patients with metastatic melanoma. So ECP seems to represent a novel independent prognostic biomarker in melanoma and perhaps other tumours as well. The measurement of this liquid biomarker in routine clinical practice would be easy and time efficient. We are, therefore, currently validating ECP as a new biomarker.

## Conclusion

Pretreatment ECP levels, collected at the time of diagnosis of stage IV metastatic disease, are associated with overall survival in melanoma patients. High serum ECP correlates with a poor prognosis, independently of the subsequent therapy.

ECP is a novel prognostic serum marker for the outcome of melanoma patients, which is independent of LDH and easy to perform in clinical practice. The negative prognostic value of high ECP level is unanticipated.

## Additional file

Additional file 1: Correlation between pretreatment ECP and LDH levels. (PNG 26 kb)

## Abbreviations

Anti-CTLA-4: Anti-cytotoxic t-lymphocyte-associated protein-4
Anti-PD-1: Anti-programmed cell death-1
CCL5: C-C motif chemokine ligand 5
CD8^+^: Cluster of differentiation 8^+^
CXCL10: C-X-C motif chemokine ligand 10
CXCL9: C-X-C motif chemokine ligand 9
ECP: Eosinophil cationic protein
EDN: Eosinophil-derived neurotoxin
ELISA: Enzyme-linked immunosorbent assay
EPO: Eosinophil peroxidase
Fig.: Figure
IgE: Immunoglobulin E
IgG: Immunoglobulin G
LDH: Lactate dehydrogenase
LPS: Lipopolysaccharides
MBP: Major basic protein
MIA: Melanoma-inhibitory-activity
MUP: Melanoma of unknown primary
OS: Overall survival
TNM: Tumour, node, metastases
