# Supplementary figures and images for: Eosinophil-cationic protein - a novel liquid prognostic biomarker in melanoma
Source: BMC Cancer. 2019 Mar 7;19:207. doi: 10.1186/s12885-019-5384-z (PMC6407264; doi:10.1186/s12885-019-5384-z)

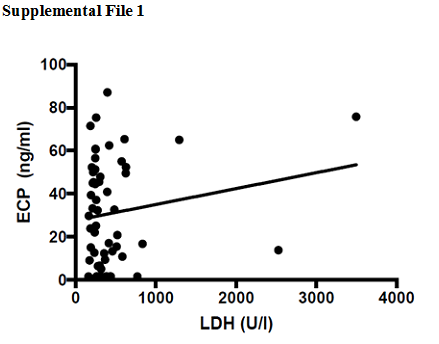

Supplement: Supplementary file 1 — Correlation between pretreatment ECP and LDH levels. (PNG 26 kb) [file 12885_2019_5384_MOESM1_ESM.png]
